# Early childhood caries: Are maternal psychosocial factors, decision-making ability, and caries status risk indicators for children in a sub-urban Nigerian population?

**DOI:** 10.1186/s12903-020-01324-y

**Published:** 2021-05-04

**Authors:** Michael Alade, Morenike Oluwatoyin Folayan, Maha El Tantawi, Ayodeji Babatunde Oginni, Abiola A. Adeniyi, Tracy L. Finlayson

**Affiliations:** 1 Obafemi Awolowo University Teaching Hospitals’ Complex, Ile-Ife, Nigeria; 2 Obafemi Awolowo University, Ile-Ife, Nigeria; 3 Faculty of Dentistry, Alexandria University, Alexandria, Egypt; 4 Innovative Aid, Abuja, Nigeria; 5 Lagos State University College of Medicine, Ikeja, Nigeria; 6 School of Public Health, San Diego State University, San Diego, CA USA

**Keywords:** General anxiety, Dental anxiety, Sense of coherence, Early childhood caries, Nigeria

## Abstract

**Objective:**

Early childhood caries (ECC) is caries in children below the age of 72 months. The aim of the study was to determine the association of maternal psychosocial factors (general anxiety, dental anxiety, sense of coherence, parenting stress, fatalism, social support, depressive symptoms, and executive dysfunction), decision-making abilities, education, income and caries status with the prevalence and severity of ECC among children resident in Ile-Ife, Nigeria.

**Methods:**

A dataset of 1549 mother–child (6–71-months-old) dyads collected through examinations and a household survey, using validated psychometric tools to measure the psychosocial factors, were analyzed. The DMFT for the mothers and the dmft for the child were determined. The association between maternal psychosocial factors, education, income, and decision-making ability, the prevalence of maternal caries, and the prevalence of ECC was determined using logistic regression analysis.

**Results:**

The prevalence of maternal caries was 3.3%, and the mean (standard deviation-SD) DMFT was 0.10 (0.76). The ECC prevalence was 4.3%, and the mean (SD) dmft was 0.13 (0.92). There was no significant difference between the prevalence and severity of maternal caries and ECC by maternal age, education, income, or decision-making abilities. There was also no significant difference in maternal caries, ECC prevalence and ECC severity by maternal psychosocial factors. The only significant association was between the prevalence of caries in the mother and children: children whose mothers had caries were over six times more likely to have ECC than were children with mothers who had no caries (AOR: 6.67; 95% CI 3.23–13.79; *p* < 0.001).

**Conclusion:**

The significant association between ECC and maternal caries prevalence suggests that prenatal oral health care for mothers may reduce the risk for ECC.

## Background

The presence of caries in young children often reflects their mothers’ caries profile [1], as children of mothers with caries tend to develop caries. This relationship may result from exposure of the child to the mother’s cariogenic diet and other caries-promoting behaviors [2]. Maternal factors associated with the risk of caries in children include maternal education [3], which can determine mothers’ knowledge and attitude to oral health [4]—including access of children to dental care [5]—and other oral-health practices [6]. Children born to young mothers were reported more likely than those born to older mothers to have more caries because of the mothers’ limited ability to handle childcare needs [7, 8]. However, the potential role of other socioeconomic status or psychosocial factors that may influence the mother’s exposure to health risks and resources to overcome stressors have not been explored.

**Fig. 1:**
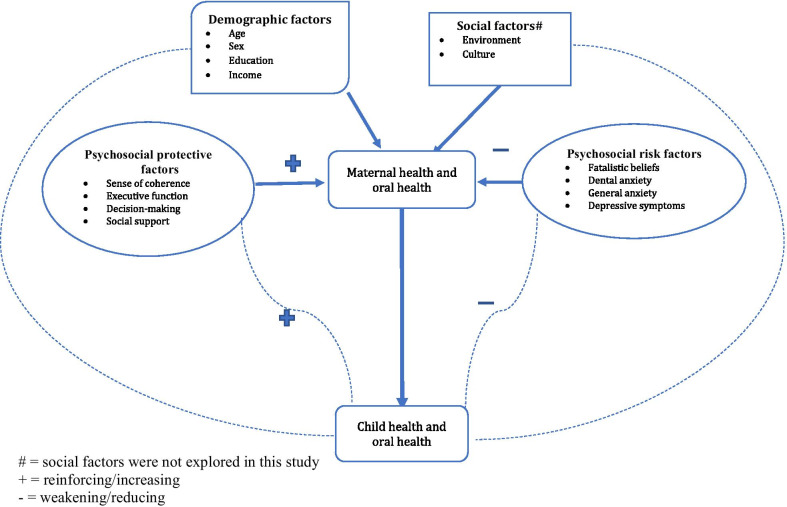
Relationship between maternal psychosocial factors and early childhood caries in preschool children

Maternal psychosocial status (general anxiety, dental anxiety, sense of coherence, parenting stress, fatalism, social support, depressive symptoms, and executive dysfunction) affects the child’s oral health, as depicted in Fig. 1. A few studies [9–12] have reported an association between maternal parenting stress and caries in children below the age of 72 months (early childhood caries or ECC) in contrast to findings of another study [13]. Maternal sense of coherence [14], anxiety [15, 16], depression [16], social support [17], and fatalism [9] also have been associated with ECC. We have found no studies that examined the relationship between maternal executive function and ECC, although this factor affects children’s health and development [18].

Executive function, also known as executive control or cognitive control, is an effortful process of using a set of mental skills to concentrate on and pay attention to getting a task done. This is know as goal-directed behavior [18], rather than relying on instinct or intuition [19, 20]. Maternal executive function is an important parenting behavior that children experience at a time when they rely on adequate caregiving [21, 22] and which is linked with child neglect [23, 24], a risk factor for ECC [25, 26]. Higher maternal executive function is associated with increased sensitivity to child needs, including their healthcare needs [21] and improved ability to cope with stress, including that associated with parenting [24].

Maternal education and income are confounders of the association between maternal psychosocial factors and children’s oral health [9, 27]. Although culture may affect the association between many factors that influence health [28], there are no studies on the association between maternal psychosocial factors and children’s caries risk in African populations; existing studies are from Western countries [29]. A factor that could be influenced by culture is the mother’s ability to take autonomous decision about her child’s access to preventive and curative healthcare services [21]. Poor utilization of dental services is a risk factor for ECC [30], and dental service utilization may be limited by the ability of a primary care giver to make that decision for her child. Maternal decision-making ability is a reflection of autonomy [31], an indication of control of resources and ideology [32], and an important phenomenon for paternalistic cultures like Nigeria where decisions are often made by the male heads of the household.

This study aims to address gaps in knowledge by examining the association between maternal caries status, psychosocial factors, and decision-making ability and the prevalence and severity of ECC in children resident in Ile-Ife, Nigeria.

## Methods

### Study design and study population

This was a secondary analysis of a dataset of 1549 mother–child dyads collected through a household survey and dental examination between December 2018 and January 2019 to determine the association between maternal psychosocial factors and ECC. The sample recruited children 6–71 months old who were living with their biological parents or legal guardians at Ife Central Local Government Area of Osun State, Nigeria and who were both at home at the time the data was collected. Using a multi-stage sampling technique. Children were excluded from study participation if they had medical conditions that could increase the risk of ECC such as HIV infection. Study participants were recruited through a multi-stage sampling technique which required sampling 10% of the 700 enumeration centers in the Local Government Area; sampling every other household on each street in the eligible enumeration area; and sampling eligible child and mother dyad in each household. The study’s sampling and recruitment methods have been reported in detail prior studies [33, 34].

### Sociodemographic profile

Maternal data obtained included mother’s age at last birthday (≤ 29 years, 30–39 years, and ≥ 40 years), education (no formal education, primary school only, secondary school only, or post-secondary school), and income (≤ 18,000 ($50) per month, 18,001–30,000 ($84) per month, 30,001–60,000 ($168) per month, and > 60,000 per month) [35]. The age of the children was recorded in months and translated into years during the data analysis. The age was dichotomized into 0–2-years-old and 3–5-years-old based on evidence that suggests different ECC risk profiles for the two age groups [36, 37].

*Maternal decision-making ability* was determined by a ‘yes’ or ‘no’ response to three questions: Which person usually decides on her healthcare? Which person usually decides on large household purchases? Which person usually decides on visits to family or relatives? [38].

### General anxiety

The 7-item Generalized Anxiety Disorder-7 scale [39], validated for use in Nigeria [40], was used to measure general anxiety. The Generalized Anxiety Disorder-7 score is calculated by assigning scores of 0–3 to the response categories, ranging from 'not at all', to 'nearly every day.' Overall scores of 5, 10, and 15 are the cut-off points for mild, moderate, and severe anxiety, respectively.

### Dental anxiety

This was measured with the Modified Dental Anxiety Scale [41], validated for use in Nigeria [42]. The scale is calculated by assigning scores of 1–5 to the response to a 5-item Likert scale questionnaire ranging from ‘not anxious’ to ‘extremely anxious.’ Overall scores range from 5–25. Scores 19 and above indicate high dental anxiety, while scores lower than 19 indicate low dental anxiety.

### Parenting stress

Six items of the 19-item Parenting Stress Index [43], used in the Detroit Dental Health Project [44] and validated for use in the Nigerian population [45], were used to measure maternal stress. Possible scores for each item ranged from 1 to 5 for responses from ‘never’ to ‘almost.’ Total scores range from 6 to 30, with higher scores reflecting more frequent experiences of stress due to parenting role [46]. Scores below the 15th percentile are regarded as low stress; 16th to 80^th^ percentile as normal stress; 81st to 84th percentile as borderline stress; and higher than the 84th percentile as high stress. For logistic regression analysis, low and normal stress levels were combined, as were borderline and high stress levels.

### Sense of coherence

This was measured with the Sense of Coherence-13 scale [47, 48] on a 7-point Likert scale adapted for use in Nigeria [49]. Possible scores ranged from 7 to 9, with higher scores indicative of a better sense of coherence. The scores were divided into lower-than-median (low coherence) and equal-to and greater-than-median (high coherence). The median score was 67.

### Depressive symptoms

The 20-item Centre for Epidemiologic Studies and Depression Scale, developed by Radloff [50] and validated for use in Nigeria [51], was used to determine the level of depressive symptoms. Each item in the scale was assigned scores of 0–3, depending on the frequency of symptoms per week, with the total score ranging from 0 to 60. Scores of less than 15 indicate no depressive symptoms; 15–21 indicate mild to moderate depressive symptoms; and 21–60 indicate major depressive symptoms. For logistic regression analysis, the scores were dichotomized into normal and mild/moderate/major depressive symptoms.

### Executive dysfunction

This was assessed with the 20-item Modified Dysexecutive questionnaire [52, 53], validated for use in Nigeria [54]. Each of the 20 statements was assigned a score of 0–4 on a 5-point scale ranging, from ‘Never’ to ‘Very often.’ Possible scores ranged from 0–80 with higher scores suggesting worsening dysfunction. For this study, executive dysfunction was dichotomized into low executive dysfunction (scores lower than the median) and high executive dysfunction (scores higher than or equal to the median). The median score was 32.

### Social support

The 12-item 7-point Multidimensional Scale of Perceived Social Support scale [55], validated for use in Nigeria [56], was used to measure perceived emotional support from family, friends, and significant others. Scores of 1.0–2.9 indicate low support; 3.0–5.0 indicate moderate support; and 5.1–7.0 indicate high support. For the logistic regression analysis, the scores were dichotomized to low/moderate and high support.

### Fatalism

The modified Powe Fatalism Inventory, which measures maternal fatalistic beliefs about ECC [57] and was validated for use in Nigeria [54], was used to measure perception of fatalism. Responses to the nine questions ranged from ‘strongly agree’ to ‘strongly disagree’ on a Likert-like scale with scores from 5–1, respectively. The scores were dichotomized to low (scores lower than the median) and high (scores higher than or equal to the median) fatalism around the median score of 25.

### Clinical examination

Five calibrated dentists with intra-examiner agreement and inter-examiner agreement Cohen’s kappa coefficient values higher than 0.80 determined the maternal and child caries status using the Decayed-Missing-Filled teeth (DMFT) index and the decayed-missing-filled teeth (dmft) index, respectively using the World Health Organisation’s criteria [58]. Caries was present if the DMFT/dmft scores were greater than 0 and absent if the DMFT/dmft scores were 0.

Children were examined either sitting on their mother’s lap or on a chair, under natural light, with plain dental mirrors. Mothers were also examined after their child, sitting on a chair under natural light, with plain dental mirrors. For both mother and the child, the teeth were not dried before examination, but gross debris was cleared with gauze where necessary. The examination of the teeth was done in an orderly manner from one tooth or tooth space to the adjacent tooth or tooth space.

#### Data analysis

The association of maternal and ECC prevalence with maternal sociodemographic characteristics (age, education and income), psychosocial status (general anxiety, dental anxiety, sense of coherence, parenting stress, fatalism, social support, depressive symptoms, and executive dysfunction) and decision-making factors was determined with Chi-square and Fisher’s Exact tests. Analysis of variance and independent sample t-test were used to compare the maternal mean DMFT and the children’s mean dmft values among and between categories of the independent factors.

Multivariable binary logistic regression analysis was used to determine the maternal factors associated with the prevalence of ECC using a series of models in line with the hierarchical theoretical model proposed by Nunes et al. [59] for determining the risk indicators for ECC, was used for developing the regression analysis for the study. Model 1 included maternal age, income and educational status and protective psychosocial factors (sense of coherence and social support). Model 2 comprised maternal age, income and educational status, and psychosocial risk factors (fatalistic belief, dental anxiety, general anxiety, depressive symptoms and executive dysfunction). Model 3 contained maternal age, income and educational status, and decision-making on health access. Model 4 comprised maternal age, income and educational status, and maternal caries. Lastly Model 5 contained all factors with *p* ≤ 0.20 from Models 1–4. The adjusted odds ratios (AORs) and their 95% confidence intervals were calculated. Multicollinearity diagnostics, with correlation matrix of coefficients, were used with each logistic model to identify independent variables that were strongly correlated (r > 0.5); in Model 3, two decision-making variables—decisions on household purchase and family visit—were dropped from the model because of multicollinearity. Statistical analyses were conducted with Stata/SE 14.0 for Windows (2015). Statistical significance was inferred at *p* ≤ 0.05.

## Results

Of the 1549 mother–child dyads recruited, 51 (3.3%) mothers and 66 (4.3%) children had caries. Maternal DMFT ranged from 0–13, with mean (SD) DMFT of 0.10 (0.76). Children’s dmft ranged from 0–19, with mean (SD) dmft of 0.13(0.92).

Table 1 shows that there was no significant difference in the prevalence of maternal and children’s caries by maternal age (*p* = 0.699), education (*p* = 0.492), income (*p* = 0.470), and decision-making status (*p* > 0.05). There also was no significant difference in the maternal and children’s caries prevalence by maternal general anxiety (*p* = 0.750), dental anxiety (*p* = 0.882), parenting stress (*p* = 0.438), sense of coherence (*p* = 0.378), social support (*p* = 0.285), depressive symptom (*p* = 0.657), and executive dysfunction (*p* = 0.215). The maternal and children’s caries prevalence was significantly lower for mothers who had high fatalism (*p* = 0.034). There was no significant difference in the severity of maternal and children’s caries by maternal sociodemographic and psychosocial factors.

**Table 1:** Bivariate association between maternal characteristics and caries in mother and child (N = 1549)

Variables (number)	Caries prevalence				Test of association (*p* value)*	Maternal mean DMFT	Child mean dmft	Test of association (*p* value)**
	Maternal (N = 51)		Child (N = 66)					
	N	%	N	%				
Age								
≤ 24 years (129)	4	7.8	4	3.1	0.094 (0.699)	0.05	0.12	0.529 (0.746)
25–34 years (919)	32	3.5	38	4.1		0.12	0.13	
35–44 years (459)	11	2.4	21	4.6		0.07	0.13	
≥ 45 years (42)	4	9.5	3	7.1		0.19	0.29	
Education status								
None (23)	2	8.7	1	4.4	0.134 (0.492)	0.83	0.22	0.001 (0.172)
Primary only (115)	6	5.2	8	7.0		0.16	0.30	
Secondary only (977)	34	3.5	41	4.2		0.09	0.13	
Above secondary (434)	9	2.1	16	3.7		0.06	0.09	
Income status								
> 18,000 per month (422)	17	4.0	20	4.7	0.494 (0.470)	0.12	0.17	0.318 (0.765)
18,000–30,000 per month (665)	17	2.6	32	4.8		0.06	0.13	
30,001–60,000 per month (417)	16	3.8	13	3.1		0.14	0.10	
> 60,000 per month (45)	1	2.2	1	2.2		0.07	0.09	
Decision-making status								
Someone else decides on access to health care					0.440 (0.353)			0.201 (0.371)
No	36	3.1	46	4.0		0.09	0.12	
Yes (394)	15	3.8	20	5.1		0.14	0.17	
Someone else decides on household purchases								
No	39	3.2	49	4.0	0.624 (0.309)	0.08	0.12	0.085 (0.289)
Yes (322)	12	3.7	17	5.3		0.16	0.18	
Someone else decides on visits to family/relatives								
No	39	3.2	50	4.1	0.737 (0.598)	0.09	0.13	0.179 (0.887)
Yes (335)	12	3.4	16	4.8		0.15	0.13	
General anxiety								
Normal (1238)	38	3.1	55	4.4	0.416 (0.750)	0.09	0.14	0.801 (0.670)
Mild (243)	9	3.7	9	3.7		0.12	0.10	
Moderate (68)	4	5.9	2	2.9		0.13	0.06	
Dental anxiety								
Low (1400)	46	3.3	60	4.3	0.964 (0.882)	0.10	0.13	0.656 (0.806)
High (149)	5	3.4	6	4.0		0.07	0.11	
Parenting stress								
Normal (1184)	34	2.9	52	4.4	0.198 (0.438)	0.10	0.14	0.937 (0.642)
Borderline (36)	1	2.8	0	0		0.06	0	
High (329)	16	4.9	14	4.3		0.10	0.12	
Sense of coherence								
Low (866)	28	3.9	34	4.8	0.206 (0.378)	0.14	0.17	0..048 (0.171)
High (683)	23	2.8	32	3.8		0.06	0.10	
Fatalism								
Low (672)	32	4.8	37	5.5	0.005 (0.034)	0.16	0.16	0.010 (0.254)
High (877)	19	2.2	29	3.3		0.06	0.11	
Social support								
Low (30)	2	6.7	3	10.0	0.202 (0.285)	0.33	0.17	0.065 (0.978)
Moderate (1264)	37	2.9	53	4.2		0.08	0.13	
High (255)	12	4.7	10	3.9		0.16	0.13	
Depressive symptoms								
Normal (1175)	40	3.4	51	4.3	0.908 (0.657)	0.10	0.13	0.936 (0.976)
Mild-moderate (207)	6	2.9	10	4.8		0.10	0.13	
Major (167)	5	3.0	5	3.0		0.12	0.12	
Executive dysfunction								
Low (866)	25	2.9	32	3.7	0.314 (0.215)	0.08	0.10	0..232 (0.133)
High (683)	26	3.8	34	5.0		0.13	0.17	

*Chi-square test/Fisher’s exact test; **ANOVA/*t *test

Table 2 shows the results of the multivariable binary logistic regression analysis for the five models. Model 1 showed that maternal age, income, and educational status and protective psychosocial factors (sense of coherence and social support) were not significantly associated with the prevalence of ECC. Model 2 showed that none of the maternal psychosocial risk factors (fatalistic belief, dental anxiety, general anxiety, depressive symptoms, and executive dysfunction) were significantly associated with the prevalence of ECC. Model 3 indicated that maternal decision-making on health access was not significantly associated with the prevalence of ECC. Model 4 indicated that maternal caries was a risk indicator for ECC (AOR: 7.38; 95% CI: 3.52–15.44; *p* < 0.001). This observation was sustained in Model 5, where children whose mothers had caries were over six times more likely to have ECC than were children whose mothers who had no caries (AOR: 6.67; 95% CI 3.23–13.79; *p* < 0.001).

**Table 2:**
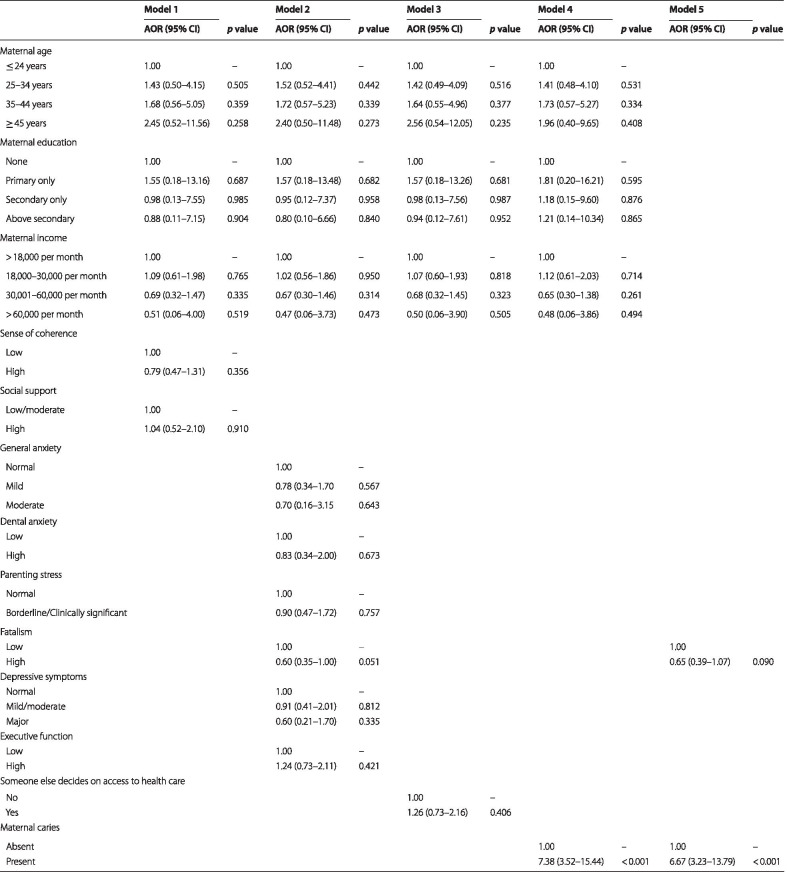
Logistic regression to determine maternal caries status, sociodemographic and psychosocial factors associated with caries status among preschool children in Ile-Ife (N = 1549)

## Discussion

Findings of the study revealed no significant association between the prevalence of ECC and maternal decision-making status and maternal socio-economic and psychosocial factors. Maternal caries status was, however, a risk indicator for ECC.

The study has several strengths: the large sample size; the sampling procedure; the inclusion of multiple independent variables in the logistic regression analysis; and the use of multiple psychosocial scales validated tools to capture the real-world experience of these mother–child dyads. Psychosocial factors reflect complex aspects of life that interact with other environmental factors to affect health. However, the R^2^ values indicate that the variables included in the study models can only explain a small fraction of the ECC risk for the children in the study population. Studies designed to determine how various aspects of the life of a child interact and contribute to the caries risk will be useful as we continue to learn about ECC mitigation strategies.

The study also had a few limitations. First, it is a cross-sectional study. As such, we cannot infer a cause-effect relationship between maternal caries status and ECC. Second, the low prevalence of ECC may have limited the ability of the study to detect other associations. Third, the study was conducted in only one of the 774 local government areas in Nigeria, so the findings cannot be generalized to Nigeria. However, the findings can be generalized to other local government areas in the country in view of the similarity in sociodemographic profiles of a sub-urban area such as Ife Central Local Government Area.

Despite the limitations, the study findings are important. First, the findings reinforce evidence that the presence of maternal caries greatly influences the child’s caries risk [60]. A risk indicator for caries in the mothers of pre-school children in the study environment is the stress of parenting [61], and the high parenting stress is a risk factor also for ECC [62, 63]. Mothers who find parenting stressful are less able to monitor and supervise their children’s oral health care and are more reactive and less proactive [63], which increases the risk of children having ECC. Higher maternal stress is associated also with greater depressive symptoms, which is a risk factor for ECC [64, 65]. This study, however, did not find an association between maternal level of parenting stress, maternal level of depressive symptoms, and the prevalence of ECC.

Maternal caries risk behaviour may therefore be the link between maternal and child caries prevalence; and not maternal psychosocial factor. The study, however, did not explore the possibility of maternal caries risk behaviour being a risk indicator for ECC. Maternal access to prenatal oral health care reduces the risk of ECC [66] as mothers thereby have access to education about healthy oral health practices for themselves and their children. There are multiple implementation studies that demonstrate that maternal access to prenatal oral health care reduces the risk of children to ECC [67, 68]. Strategic interventions found to be safe needs to be scaled up through a maternal-child care program [67]. In a systematic review of maternal risk factors, evidence suggested prenatal education and interventions were effective to reduce bacteria transmission from mothers to children [69]. An implementation research to explore the effect of prenatal oral health care as a risk mitigator for ECC may help further reduce the low prevalence of ECC in the community to one that is close to elimination [70].

Unlike prior studies which had found maternal age [7, 8] and education [3] as risk factors for caries in children, this study did not demonstrate an association between the prevalence of ECC and these factors, nor was there a significant association between maternal income level and the prevalence of ECC. It also did not find a significant association between the decision-making status of the mother the prevalence of ECC though the prevalence of ECC was higher in children whose mothers had someone else decides on access to health care for them. A prior study had indicated that maternal empowerment and ability to make decision about self-access to health care may not be protective for ECC if there is no social support for women who are the primary carers for young children to continue to provide child care when they go back to work to make income (many parameters of empowerment for women is measured as economic empowerment) [71]. Further studies are needed to understand if and how maternal decision-making ability may influence the risk of children to ECC.

Of interest is the low prevalence of caries in both the mother and child. Although this is not the focus of the study, this finding is important because of the potential for non-differential error in the inter- and intra-examiner reliability Kappa coefficients which may assumably introduce measurement bias and an increase in type error 2. Prior studies had however indicated that the prevalence of ECC and caries in adults in the study population are low [72–75]. The reason for this is unknown but a suggestion is the access to fluoridated toothpaste: the food and drug regulatory agency only approves fluoride containing toothpaste for sale in Nigeria and as high as 95% of the study population use fluoridated toothpaste [72]. Ile-Ife is also a semi-urban agrarian community where consumption of refined carbohydrates and processed food is low. These postulated reasons for the low caries prevalence in the study population needs to be studied further. Also, because of the low caries prevalence, future studies on the predictors of ECC in the same population should use case–control design though the use of logistic regression for inferential analysis was appropriate for the low caries prevalence.

We observed that the prevalence of ECC was higher than the prevalence of caries in mothers. The higher prevalence of caries in the children than in the mothers may indicate a growing oral health problem for children in the community. Kubota et al. [76] had speculated of this possibility in the study population when they noted that an increase in the consumption of refined carbohydrates co-exiting with poor oral hygiene will greatly increase the incidence of caries which was low at the time of their study. Though high consumption of refined carbohydrates in-between-meals and fair oral hygiene increase the risk for ECC in the study population [72], between 2014 and 2019, the proportion of those who consume refined carbohydrate had decreased (30.6% vs 14.0%), there had been an increase in the proportion of children who have poor oral hygiene (2.2% vs 3.9%) and the prevalence of ECC has reduced (6.6% vs 4.3%) [72, 77]. Further studies are required to understand the intergenerational differences in the prevalence of caries observed in this study.

## Conclusion

Although we found no significant association between ECC and maternal decision-making status and socio-economic and psychosocial factors, we found an association between ECC and maternal caries status. This finding suggests that infants, toddlers, and preschool children in the study population would benefit from a prenatal maternal oral care program that reduced the risk of ECC in other populations. The link between maternal and child caries status in the study population needs to be studied further.

## Data Availability

The data used for this study are presented in the study. The primary study from which data was extracted for this study is not yet published. Data can however be accessible on request.
